# Cajal and the Spanish Neurological School: Neuroscience Would Have Been a Different Story Without Them

**DOI:** 10.3389/fncel.2019.00187

**Published:** 2019-05-24

**Authors:** Fernando de Castro

**Affiliations:** Grupo de Neurobiología del Desarrollo – GNDe, Instituto Cajal (CSIC), Madrid, Spain

**Keywords:** neuron, oligodendrocyte, microglia, synapse, Santiago Ramón y Cajal, Nicolás Achúcarro, Pío del Río-Hortega, Rafael Lorente de Nó

## Abstract

Santiago Ramón y Cajal was still young when he came across the *reazione nera*, discovered by the Italian Camillo Golgi. Cajal became absolutely entranced by the fine structure of the nervous system this technique revealed, which led him to embark on one of the last truly epic endeavors in Modern History: the characterization of nervous cells, and of their organization to form the brain. Cajal remained in Spain throughout his scientific career, working for years alone. With international recognition, Cajal began recruiting brilliant students as collaborators. A handful of his pupils also made decisive discoveries that served to lay the foundations of modern Neuroscience. Cajal’s brother Pedro, Tello, Domingo Sánchez, Achúcarro, Lafora, Río-Hortega, de Castro and Lorente de Nó worked side by side with *El Maestro*. While Cajal himself pronounced some of the basic rules that have helped us to understand the nervous system (the neuron theory, the law of dynamic polarization of the neuron), as well as providing innumerable details about the histological organization of the different neural structures, it was Pío del Río-Hortega who identified two of the four main cell types in the CNS (oligodendrocytes and microglia), and Fernando de Castro who described the innervation of the blood vessels and identified the first chemoreceptors in the carotid body. Together, this group of scientists is known as the Spanish Neurological School, and if they had not existed, the History of Neuroscience would surely have been quite a different story; and proof that Cajal was a truly exceptional scientist but he was not an exception for Spanish Science.

## Introduction

Santiago Ramón y Cajal was born in 1852 in Petilla de Aragón, a small village in the North of Spain, in the foothills of the Pyrenees. His origins and his studies at the Medical School of Zaragoza did not provide any indication that he was destined to follow an outstanding scientific career, making fundamental contributions that were to establish the pillars on which modern Neurology and Neuroscience have been founded. Details from the early life of the young Cajal^1^ can be found in many publications, including his autobiography (Ramón y Cajal, 1923^2^; De Carlos and Pedraza, 2014; Alonso and De Carlos, 2018) and so, we shall skip this period of his life and the years he spent as a doctor in the Spanish Army, both during the last Carlist war and the war in Cuba^3^. Cajal returned to Spain from Cuba in 1875 with a severe illness and once he had recovered in a small hospital in Zaragoza, he went to Madrid to work on his doctorate. It is in Madrid that the still young Ramón y Cajal met professor Aureliano Maestre de San Juan (1828–1890), the first chair of Histology and Pathology in Spain. Maestre de San Juan introduced Cajal to his first histological slides, a pivotal moment for the young doctor. Indeed, the interest in general animal histology he instilled in Cajal and the latter’s work on bacteriology and inflammation, were fundamental for Santiago Ramón y Cajal to obtain his first full professorship in Human Anatomy at the University of Valencia (1883). It was during this period that Cajal met Luis Simarro (1851–1921), who was firmly established in Madrid (López-Piñero, 2007). Indeed, it was in the laboratory that Simarro had set-up at his home in the street now known as “Augusto Figueroa” where Cajal first discovered slides of nervous tissue treated with the *reazione nera* (after the impregnation and the chrome-silver reaction, random cells become black), a technique developed by Camillo Golgi (1843–1926), the histologist from Pavia (Italy). As such, these two figures, Maestre de San Juan and Simarro, each played a fundamental role in guiding the career of Santiago Ramón y Cajal, the former luring him into the world of Histology and the latter, introducing him to the Golgi method. Together, these two figureheads established the basis for one of the most successful scientific careers in the History of Science. After Simarro introduced him to Golgi’s new technique, Cajal immediately began to work with and master this technique, modifying it and improving the results obtained, and ultimately embarking on a journey into relatively unknown territory: the fine structure of the central nervous system (CNS).

## Cajal’s Golden Era at the University of Barcelona

**FIGURE 1:**
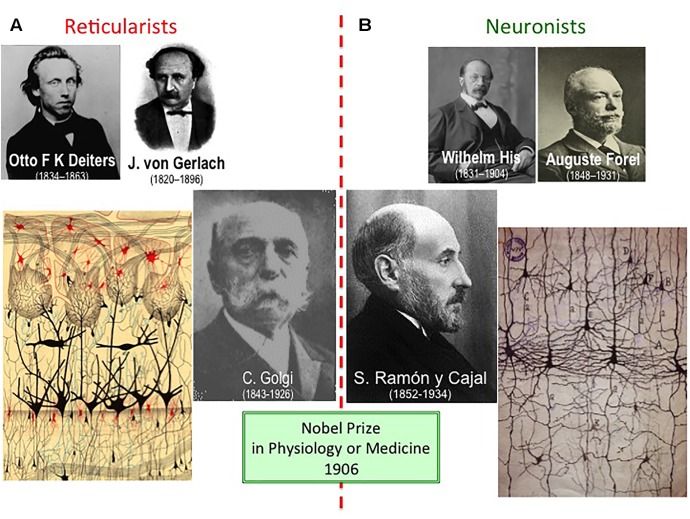
Reticularists vs. Neuronists. **(A)** The “reticular theory” was originally raised by Otto Reiters and Joseph von Gerlach, although the most identifiable reticularist at the beginning of the XXth century was Camillo Golgi. **(B)** “Neuronism” was initially proposed by Wilhelm His and August Forel as the “free endings hypothesis,” yet with the appearance of Santiago Ramón y Cajal in the field, he became the undoubted leader of the “neuron theory.” The way Golgi (drawing on the left: olfactory bulb) and Cajal (idem on the right: pyramidal neurons from the cerebral cortex) illustrated their findings clearly differed, although both finally shared the Nobel Prize 1906. This was the very first time that the Nobel Prize in Physiology or Medicine was shared by two laureates, something relatively common nowadays; previously, two laureates shared the Nobel Prize in Peace (1901 and 1902), and in Literature (1904), and three did it in Physics (1903).

In 1888, Santiago Ramón y Cajal was named as Chair of Histology and Pathology at the University of Barcelona, the second most important department in Spain at that time, and more widely connected to the rest of Europe than that in Valencia. It was in Barcelona where Cajal perfected and became a real master of the silver impregnation technique he had indirectly learned from Golgi. Not in vain, Cajal himself considered 1888 as *“mi año cumbre, mi año de fortuna”* (“my summit year, my year of fortune”: Ramón y Cajal, 1923). As a result, he performed systematic studies on the microscopic structure of the brain of birds and small mammals. These studies led him to question the most dominant theory regarding the organization of the nervous system, the “reticular theory” attributed principally to Otto Deiters (1834–1863) and Joseph von Gerlach (1820–1896), which proposed that the components of the nervous system formed a continuous syncytial network (Figure 1: Shepherd, 1991). At the end of the XIXth century, the main flag bearer of the reticular theory was Camillo Golgi (1843–1926) and paradoxically, it was through the technique developed by the Italian histologist that Cajal was able to suggest an alternative way to explain how the nervous system was organized: the so-called “neuron theory,” after the fortunate name “neuron” coined by Waldeyer in 1891 (Shepherd, 1991).

**FIGURE 2:**
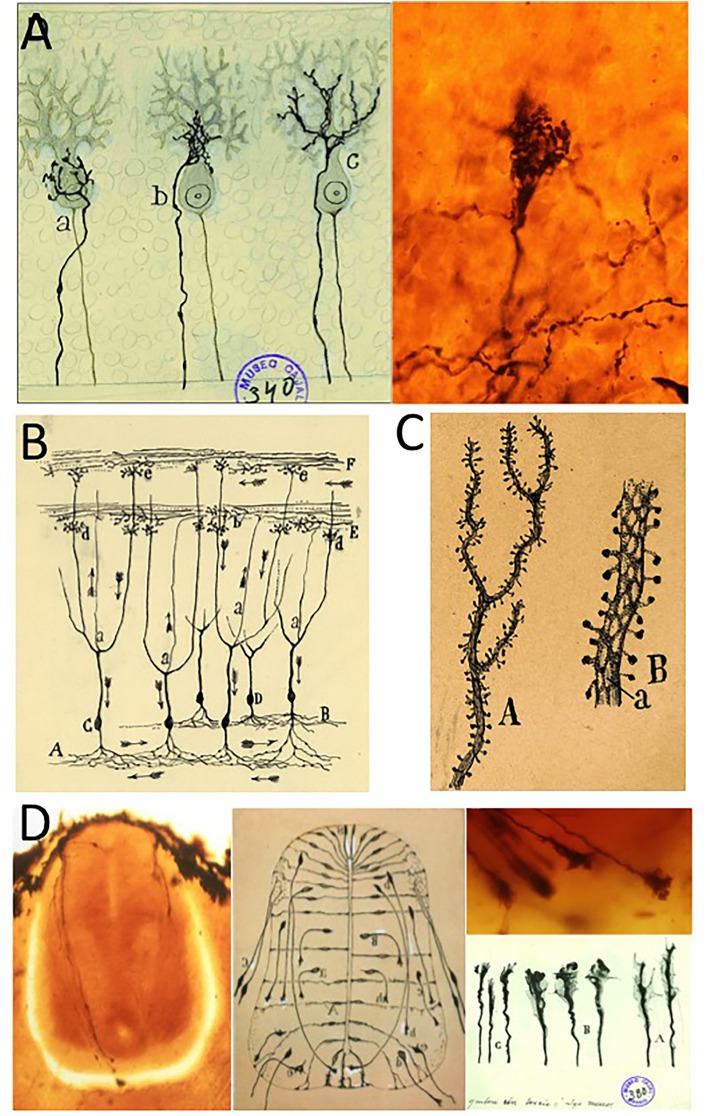
The main discoveries of Santiago Ramón y Cajal. **(A)** Original drawing from Cajal (published in Ramón y Cajal, 1888) and a modern photograph taken from a Cajal’s original histological slide illustrating the climbing fibers freely ending on a Purkinje cell (adapted from de Castro et al., 2007). **(B)** Cajal’s scheme illustrating the “dynamic polarization of neurons,” in which the arrows show the direction of the nerve impulses in the structure. **(C)** Original drawing to illustrate dendritic spines, originally published in 1890 (modified from Yuste, 2015). **(D)** Modern photographs from original histological slides of Cajal, the embryonic spinal cord of the chicken (and axonal growth cones in detail), and Cajal’s original schemes illustrating both the commissural neurons with long axons and the growth cones at their tips. Photos of different growth cones were taken as if different photograms of a moving picture (adapted from de Castro et al., 2007; with permission from Elsevier-Brain Research). The modern photographs in panels **(A,D)** were taken by Dr. Juan A. De Carlos (Instituto Cajal-CSIC; Madrid, Spain). Original schemes shown in panels **(B,C)** belong to the Fernando de Castro Archive (Censo-Guía de Archivos de España e Iberoamérica #ES.28079.AFC; Madrid, Spain), which is part of UNESCO’s World Heritage (Memory of the World International Register) as a part of the “Archives of Santiago Ramón y Cajal and the Spanish Neurohistological School” (http://www.unesco.org/new/en/communication-and-information/memory-of-the-world/register/full-list-of-registered-heritage/registered-heritage-page-1/archives-of-santiago-ramon-y-cajal-and-the-spanish-neurohistological-school/).

Santiago Ramón y Cajal’s “neuron theory” proposed that the nervous system was made up of microscopic cells (neurons), each independent from one another but that establish complex patterns of connections (Figure 2A). Cajal studied his histological slides in great detail and in 1891 he proposed the law of “dynamic polarization,” establishing the information flow within each neuron that was generally oriented from the dendrites toward the axon (Figure 2B,C: Ramón y Cajal, 1923; de Castro, 1981). This theory was illustrated by arrows that indicated the pathway followed by nerve impulses. Both these theories have since been confirmed once and again as the Neurosciences have evolved.

In these years of solitary work, Santiago Ramón y Cajal obtained a lot of useful data from the study of the CNS of very young animals, and even that of embryos of different species. He baptized this approach the “ontogenetic method”, which took advantage of the fact that the Golgi method was more effective on the nervous tissue of embryos because: (i) there are fewer cells with and they have simpler ramifications than in adults; and (ii) the myelin sheaths are not yet properly formed, allowing the silver impregnation to reach the neurons more easily (de Castro et al., 2007). Cajal’s ontogenetic method was fundamental for the Spanish histologist to describe a structure at the tip of the axons, an element he named the “axonal growth cone” (Ramón y Cajal, 1890). This first description of the growth cone came from studies of the commissural cells in the spinal cord of chick embryos (Figure 2D). The unpredictable forms of these fascinating structures persuaded Cajal that they were used by axons to interpret the chemical signals present in their milieu. Indeed, just a few months later Cajal proposed the “chemotropic hypothesis,” based on the presence of gradients of molecules that selectively attract or repel growing axons, guiding them toward their final targets where they contact and form synapses^4^ with other neurons (Ramón y Cajal, 1893; de Castro, 2003; Tamaríz and Varela-Echevarría, 2015). It was through the dissection of the chick embryo spinal cord that Cajal was able to describe axon growth cones. However, it took almost a century for Marc Tessier-Lavigne to identify the first chemotropic molecule, confirming Cajal’s chemotropic hypothesis and opening new perspectives in Developmental Neurobiology, soon to be incorporated into the field of Neurology as a whole (Tessier-Lavigne et al., 1988; Tessier-Lavigne and Goodman, 1996; de Castro, 2003).

The scientific theories Santiago Ramón y Cajal established served as the foundations upon which he launched his exploration of almost every structure in the CNS. These efforts largely involved extensive comparative studies (using material from humans, dogs, cats, rodents, birds, reptiles…), which led him to discover novel nuclei and cell types, and to reorganize the ideas regarding the connections between neural regions and nuclei. By the time all these discoveries and descriptions had been compiled into a single volume, published under the title “Textura del sistema nervioso del hombre y los vertebrados” (Ramón y Cajal, 1899), Cajal was already internationally recognized as a reputed anatomist and histologist. It was Albrecht Von Köelliker (1817–1905), the mighty Swiss anatomist and histologist (professor at Wurzburg), who first recognized the value of Cajal’s discoveries when he came across them at the congress of anatomists held in Berlin (1889). His astonishment when contemplating the histological slides that had arrived from the far South-Western corner of Europe, was what drove Köelliker to ensure that Cajal received the recognition he deserved. Consequently, offers to translate his huge opus magnum into other languages were immediately forthcoming (French, English, German…), spreading this new view of the structure and organization of the nervous system across the globe. And then came the award, some of those most transcendental in the scientific world, including: the Moscow Prize (1900); the Helmholtz Medal (1905) from the German Leopoldina Imperial Academy; and ultimately, the relatively recently established Nobel Prize (1906). As a consequence of this international recognition, the Spanish authorities and society rapidly recognized the titanic scientific work and stature of Santiago Ramón y Cajal.

## The Nobel Prize in Medicine and the Foundation of a True School

**FIGURE 3:**
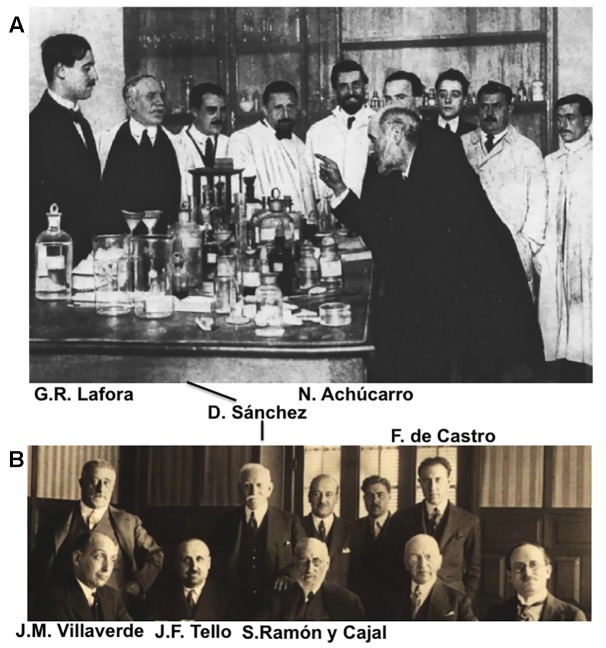
Cajal and some members of the Spanish Neurological School. **(A)** Picture taken circa 1915 at the Laboratorio de Investigaciones Biológicas (Madrid, Spain), where Santiago Ramón y Cajal simulates a lesson to his disciples and technicians (to the general delight of the former). From the left to the right, Gonzalo R. Lafora (first), Domingo Sánchez (second), and Nicolás Achúcarro (fifth) can be identified. **(B)** A banquet in honor of two foreign scientists (the Polish Prof. Szymonowski and the French Prof. May –both to the right of Cajal in the picture), celebrated in Madrid in the 1930s. Francisco Tello, Domingo Sánchez and Fernando de Castro are all standing at the back. Jorge Ramón Fañanás (son of Cajal) is just behind his father and at the right of Domingo Sánchez.

The international award Santiago Ramón y Cajal received for his scientific achievements led the king of Spain, Alfonso XIII, to build him a laboratory in Madrid, the so-called “Laboratorio de Investigaciones Biológicas” (Figure 3A). This was a fully equipped, modern (for the time) laboratory suitable for a leading anatomo-histologist, and with the capacity to hire a series of scientific collaborators, the first of whom was Jorge Francisco Tello (1880–1958; see below). The king also prompted the government to establish a body to fund promising young scientists and artists, called the “Junta de Ampliación de Estudios” (JAE) and that was presided by Cajal. This organism was established to enable these talented professionals to study abroad, to learn from the leaders in their field in other countries and to then return to Spain to further develop their careers. As such, Cajal’s international prestige and the economic support they received gave a new generation of scientists all they needed to explore their own creativity. In turn, Cajal was able to attract brilliant disciples and collaborators to his laboratory, who not only learned directly from the Maestro but as a result, formed what is now known as the Spanish Neurohistological School or more familiarly, the Cajal School or the School of Madrid: considering the scope of their scientific discoveries, far beyond pure structure and histology of the nervous system, the name Spanish Neurological School seems more suitable (and therefore, the one we will use along this current text).

Although Cajal’s had a long list of collaborators throughout his career (Figure 3: reviewed in De Carlos and Pedraza, 2014), the more constant and outstanding collaborators constitute what is universally recognized as one of the most transcendental and genuine examples of a scientific school in the field of Biomedicine, perhaps alongside that formed around the great French microbiologist Louis Pasteur (1822–1895). These names include Francisco Tello, Pedro Ramón y Cajal (the younger brother of Santiago^5^), Domingo Sánchez, Nicolás Achúcarro, Pío del Río-Hortega, Fernando de Castro and Rafael Lorente de Nó. This review aims to focus on these disciples and collaborators of Cajal, and far from exhaustively reflecting their life and scientific achievements, they will be considered in reference to their scientific contributions and how these were related to the work of their Maestro.

## The Neuroanatomical and Neurohistological Descriptions of Cajal’s Disciples

It is recognized that Santiago Ramón y Cajal provided a particularly important and revolutionary contribution to understanding the structure of the nervous system. As a result, all his disciples were trained in the study of Neuroanatomy and Neurohistology, and they also made some important contributions to this field as well. For example, Francisco Tello studied the structure of the pituitary gland and the lateral geniculate nucleus, before moving to the chicken embryo to describe neurogenesis during different stages of development (Ramón y Cajal, 1923; de Castro, 1981; Andrés-Barquín, 2002; Martínez-Tello, 2019). Significantly, these studies have received further recognition in recent years, with Francisco Tello now being considered as the only disciple of Cajal who carried out important research in the field of neuroembryology (mainly in the 1920–1930s: Puelles, 2009).

Both Pedro Ramón y Cajal (1854–1950) and Domingo Sánchez (1860–1947) studied the CNS of different species of invertebrates and vertebrates: the first performed interesting comparative studies on Amphibians, Reptiles and Birds, while the second was mainly focused on insects (Ramón y Cajal and Sánchez, 1915). This may be considered logical for a naturalist like Domingo Sánchez, who was relatively old when he started working with Cajal after a career as a zoologist and naturalist in the Philippines. As recognized by Cajal himself, the contributions of his younger brother Pedro Ramón y Cajal were also fundamental for him to be convinced about what was maybe his most relevant contributions, the Neuron Theory and the Law of Dynamic Polarization of neurons (see above): *“In the conclusions of my work, I described accurately the route that the visual current takes, confirming the opinions of my brother”* (Ramón y Cajal, 1923). Perhaps the same can also be applied to Domingo Sánchez (Andrés-Barquín, 2002).

**FIGURE 4:**
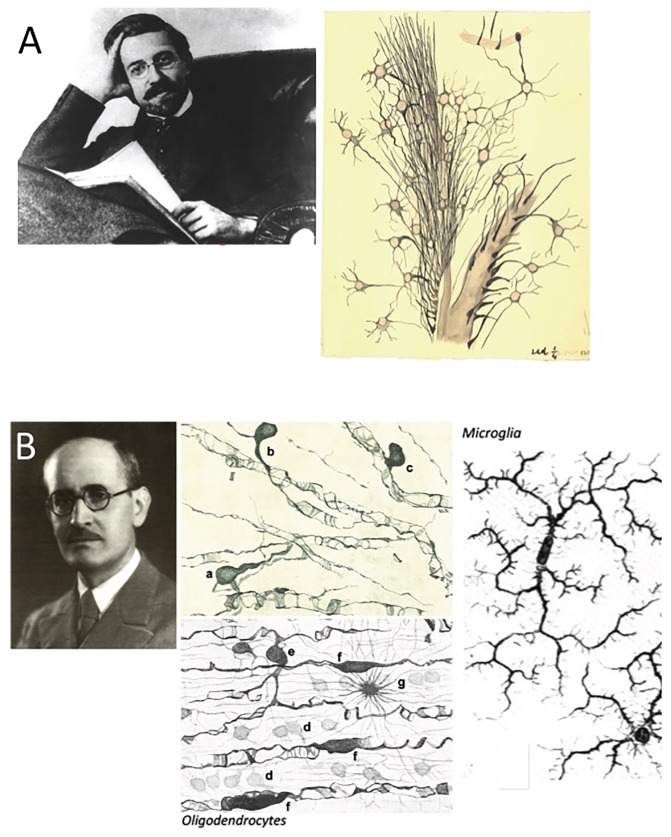
Nicolás Achúcarro and Pío del Río-Hortega. **(A)** Achúcarro at the height of his intellectual maturity and one of his original drawings illustrating neuroglia in the Ammon’s horn of a monkey, dated 1914-15. **(B)** Pío del Río-Hortega and drawings (from the left to the right) of oligodendrocytes and microglia (adapted from Sierra et al., 2016 with permission from Glia). The original drawing in panel **(A)** and the portrait in panel **(B)** belong to the Fernando de Castro Archive (Censo-Guía de Archivos de España e Iberoamérica #ES.28079.AFC; Madrid, Spain), which is part of UNESCO’s World Heritage (Memory of the World International Register) as part of the “Archives of Santiago Ramón y Cajal and the Spanish Neurohistological School” (http://www.unesco.org/new/en/communication-and-information/memory-of-the-world/register/full-list-of-registered-heritage/registered-heritage-page-1/archives-of-santiago-ramon-y-cajal-and-the-spanish-neurohistological-school/).

In an initial collaboration that was cut short by the premature death of the former, Nicolás Achúcarro (1880–1918) and Pío del Río-Hortega (1882–1945) dissected out the components of the so-called “third element” in the CNS (Figure 4; for a recent review in this subject see: Pérez-Cerdá et al., 2015; Tremblay et al., 2015). After a training period that took him to several of the main laboratories in Europe, and 2 years in the United States organizing the mental health service in Washington DC, the neuropathologist Achúcarro began to study neuroglia and granuloadipose cells (Achúcarro, 1909). However, it was Pío del Río-Hortega, his collaborator and successor as the head of the “Laboratorio de Neuropatología” (the branch of Cajal’s laboratory specifically dedicated to the study of neuropathologies), who identified these as microglia and as a colophon or oligodendroglia. While the microglia are the resident macrophages in the CNS, with a mesodermal but not neuroectodermal origin, the oligodendroglia he identified in the CNS were later defined as the Schwann cells (del Río-Hortega, 1919a,b,c, 1920, 1921; for an annotated translation of these works in English see Sierra et al., 2016). These discoveries by Río-Hortega were clouded in the personal side because of his dispute with Cajal. The dispute apparently burst when Cajal suggested the relevance of Robertson in the identification of microglia (maybe the clearest and most complete description of the dispute can be found in: Sierra et al., 2016 –with several other interesting details-), although different pertinent testimonies strongly suggest that the strong personality of the Maestro was used by a third person to make him clash against his brilliant collaborator: as a consequence, Cajal expulsed Río-Hortega from the laboratory, but ordered to build a brand new lab at the Residencia de Estudiantes, funded by JAE, as well as continued publishing scientific works from Pío del Río-Hortega in the journal founded and directed by Cajal. With time, both gigantic neuroscientists became friends, again and them forgot this sad and obscure episode (de Castro, 1981; Sierra et al., 2016).

**FIGURE 5:**
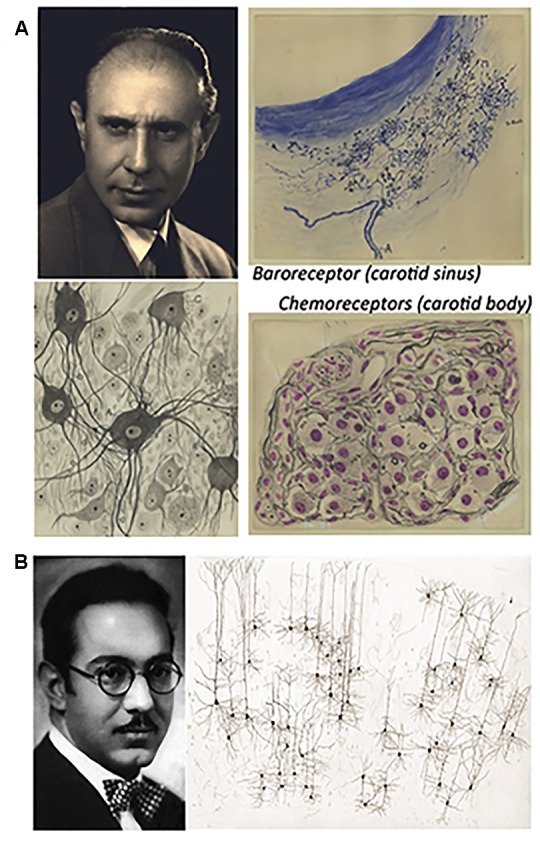
Fernando de Castro and Rafael Lorente de Nó. **(A)** Portrait of Fernando de Castro before the Spanish Civil War and his original drawings of sympathetic neurons (in the center), a baroreceptor from the carotid sinus (upper right) and chemotropic receptors in the carotid body (lower right). **(B)** Rafael Lorente de Nó, 1931, when he moved to the United States, where he remained for the rest of his career, and one of his original drawings illustrating the columnar organization of the cerebral cortex (adapted from Larriva-Sahd, 2014). Partial content of panels **(A,B)** have been reproduced with permission from Frontiers Media. All the images included in panel **(A)** belong to the Fernando de Castro Archive (Censo-Guía de Archivos de España e Iberoamérica #ES.28079.AFC; Madrid, Spain), which is part of UNESCO’s World Heritage (Memory of the World International Register) as part of “the Archives of Santiago Ramón y Cajal and the Spanish Neurohistological School” (http://www.unesco.org/new/en/communication-and-information/memory-of-the-world/register/full-list-of-registered-heritage/registered-heritage-page-1/archives-of-santiago-ramon-y-cajal-and-the-spanish-neurohistological-school/).

The youngest direct disciples of Cajal, Fernando de Castro (1896–1967) and Rafael Lorente de Nó (1902–1990), also made relevant neuroanatomical contributions (Figure 5). Fernando de Castro centered his interest on the different structures in the peripheral nervous system, such as the sympathetic and parasympathetic ganglia (Figure 5A: de Castro, 1921, 1923b; for a recent review on this subject see: de Castro, 2016; Ros and de Castro, 2019), becoming a worldwide authority. Indeed, the neurosurgeon and neuropathologist Wilder S. Penfiend trusted him to write the chapters on these structures in his famous treatise (de Castro, 1932a,b), and on the innervation of the pancreas (de Castro, 1923a). Undoubtedly his most famous and best recognized of his works was the first description of blood chemoreceptors in the carotid bodies, responsible for the cardio-respiratory reflexes. Indeed, this contribution helped the Belgian physio-pharmacologist Cornelius Heymans to obtain the Nobel Prize in Physiology or Medicine 1938 (Figure 5A: de Castro, 1926, 1928, 1981; for recent reviews see de Castro, 2008, 2009a,b; González et al., 2014; Ros and de Castro, 2019).

Rafael Lorente de Nó was as an extraordinary histologist (Larriva-Sahd, 2014) and he was a reputed neuroanatomist in his younger years, before becoming a leading figure in the field of Neurophysiology (Figure 5B, and see below). His training with Cajal helped him describe the anatomical organization of the acoustic-vestibular system, and opened the door for him to work with Oskar and Cécile Vogt, and with the 1914 Nobel Prize laureate in Physiology or Medicine, Robert Barany (1876–1936).

## The Cajal School on Neuropathologies

Although Santiago Ramón y Cajal mainly focused his descriptions on the normal structure of the nervous system and its development, he also explored the degeneration of neural cells after damage (significantly, neurons), as well as their potential regeneration (Ramón y Cajal, 1905, 1906^6^). Indeed, Cajal was perhaps the first to suggest the plasticity-related nature of Alzheimer’s disease^7^, attempting to mimic the pathology of Alzheimer’s disease as an experimental paradigm. These original findings were later collated and complemented with the contributions of others in a volume that Cajal titled “Studies on the degeneration and regeneration of the nervous system” (Ramón y Cajal, 1913). In this volume, he incorporated relevant contributions from his first true disciple, Jorge Francisco Tello, who at that time was employing silver staining to elegantly describe the re-enlargement of neurofibrils after hibernation in reptiles (Tello, 1903, 1904). Tello produced some pioneering descriptions of degeneration and regeneration using nerve transplants, which have received renewed attention in the last decade, as well as studying the influence of neurotropism on the regeneration of neural structures (Tello, 1907, 1911, 1914). Indeed, his colleague Fernando de Castro did not hesitate to declare Jorge Francisco Tello as “the best paladin” in support of the Cajal’s neurotropic hypothesis (de Castro, 1981).

Similarly, Fernando de Castro’s descriptions of the human sensitive and vegetative ganglia under pathological circumstances cannot be separated from those under normal conditions, and many of his conclusions from these comparative normal-pathological studies lead him to be considered a world authority in this field (de Castro, 1921, 1932a,b). All three, Cajal, Tello and de Castro were professors of normal Histology and Pathology at the Universidad Central of Madrid (now known as the Universidad Complutense de Madrid). As indicated above, Fernando de Castro attempted to incorporate information obtained by the brilliant Italian histologist Giuseppe Levi (1872–1965) through the *in vitro* culture of nervous tissue/cells into his own work on the spontaneous regeneration of peripheral nerves. This formed the basis for de Castro’s physiological studies to demonstrate the nature of arterial chemoreceptors, even after Heymans had already been awarded the Nobel Prize for this in 1938 (de Castro, 1933, 1934, 1937, 1942; for a review, see: de Castro, 2009b).

The brilliant neuropathologist Nicolás Achúcarro (1880–1918), one of the most reputed experts in his field at that time (Pierre Marie, Babinsky, Tanzi, Lugaro, Alzheimer and Kraepelin), joined Cajal’s laboratory as the director of the Laboratory of Neuropathology in 1913 in order to boost the study of pathologies. Besides his contribution to the description of microglia (see above), Achúcarro was the first to describe the neuropathological changes provoked by rabies’ virus infection, as well as the changes to the brain’s blood vessels and the transformation of normal astroglia into reactive astrocytes in progressive general paralysis. His studies complemented the initial descriptions of his former advisor, Alzheimer, with a detailed description of the modifications suffered by astrocytes in senile dementia (Achúcarro, 1909; Achúcarro, 1911a; Achúcarro, 1913; Achúcarro and Gayarre, 1914). In contrast to the classic view of Virchöw, Achúcarro was the first to suggest that glial malfunction could itself generate disease in the CNS, without primary damage to neurons (Achúcarro, 1913). This is a concept that is re-emerging today in order to differentiate changes in glial cells, “gliopathies,” to those that follow primary damage to neurons or “neuropathies” (Verkhratsky et al., 2012; Tremblay et al., 2015).

When requested by the American authorities to organize and head a mental health service in Washington DC in 1908 (the Government Hospital for the Insane), the highly regarded German neuropathologist Alois Alzheimer recommended his young Spanish pupil, Nicolás Achúcarro, as the most apt person for the job. Indeed, Achúcarro worked for 2 years at this post, establishing a department that eventually became a fundamental part of what we know as the National Institutes of Health (NIH). Far from being the only Spanish contribution to this institution, when Achúcarro returned to Spain in to work with Cajal, his successor in Washington was another brilliant Spanish neuropathologist, Gonzalo Rodríguez-Lafora (1886–1971), who had trained with Oskar Vogt, Minkowski, Kraepelin and Alzheimer. It was during his stay in the United States, before joining Cajal’s laboratory in 1912, when Lafora described the disease that took his name: the “familial progressive myoclonic epilepsy with intraneuronal presence of amyloid bodies” or Lafora’s disease (Lafora and Glueck, 1911)^8^. Curiously, this is the only neurological disease named after a Spaniard, because although the association of anosmia and hypogonadotropic hypogonadism was first described by one of Cajal’s mentors, Maestre de San Juan (1856), it is commonly known as Kallmann’s syndrome after the German-born neuropsychiatrist who described the genetic association of this disease in 1944 (for details see de Castro et al., 2017).

Lafora’s experiences in the United States were quite intense, as he was one of the first pathologists to study the pathology of Alzheimer’s disease after its initial description by Alzheimer, as well as studying the general reactivity of the brain to insult (Lafora, 1911a,b,c). Once back in Spain, he became very active in medical and intellectual spheres. In 1920 he founded and became director of the journal “Archivos de Neurobiología,” together with perhaps the most influential Spanish philosopher of the last two centuries, José Ortega y Gasset and the psychiatrist José Miguel Sacristán. Santiago Ramón y Cajal, Río-Hortega and Luis Simarro were among the most relevant contributors to the journal, which was closed by the authorities in power at the end of the Spanish Civil War in 1939. Finally, the Spanish League for Mental Health was founded in 1926, with Santiago Ramón y Cajal as its first President and Gonzalo Rodríguez-Lafora its Vice-president (López-Muñoz et al., 2008).

As a culmination of the contribution of the Spanish Neurological School to Neuropathology, Pío del Río-Hortega was a pioneer in the field of histopathology and especially, in terms of the cytopathology of nervous system tumors (essentially with the help of silver staining methods). Indeed, he proposed the first classification of nervous system cancers, based on cell lineage and the developmental origins of the transformed cells (del Río-Hortega, 1932, 1934; there is an English version of the classification: del Río-Hortega, 1962; for a recent review see Ramón y Cajal Agüeras, 2016). Although Río-Hortega’s first thoughts on neural tumors were published in the early 1910s (del Río-Hortega, 1911, 1912), it was between 1928 and 1936 when his name became universally recognized in this area (this was his main research subject until his death), when he was already known for the identification of oligodendroglia and microglia (see above). His cytological classification of nervous system tumors was controversial at that time (del Río-Hortega, 1932, 1933), yet it was fundamental to complement that proposed by Bailey and Cushing, with important contributions by Penfield (who actually went to Spain in the mid-1920s to work with Pío del Río-Hortega for a relatively short but fundamental stage of his training). Indeed, Río-Hortega’s classification was accepted when he presented it at the International Cancer Congress for the Scientific and Social Fight Against Cancer (Madrid, 1933), where he campaigned for a worldwide harmonization of the terminology used in the study and treatment of nervous system tumors (exquisitely summarized in: Ramón y Cajal Agüeras, 2016). In fact, his studies and classification of oligodendrogliomas, and of tumors in the optic chiasm and nerves are particularly remarkable (del Río-Hortega, 1944a,b), especially as they were carried out in his last years and in exile, before ironically dying as a consequence of cancer.

## The Neurophysiological Orientation of Cajal’s School

Undoubtedly, one of the main contributions of Santiago Ramón y Cajal had an unambiguous physiological meaning, the so-called law of the “dynamic polarization of neurons” that was first presented and finalized between 1891 and 1897 (Figure 2B: Ramón y Cajal, 1923; Shepherd, 1991; Delgado-García, 2015). This was the very first serious attempt to understand how nervous information travels and it represented the first step in unraveling the physiology of the nervous system, with Cajal being the first to produce accurate diagrams of the reflex pathways (Ramón y Cajal, 1890). Based on his description of synapses, Santiago Ramón y Cajal proposed that the selective strengthening of specific synapses could underlie learning (Ramón y Cajal, 1895). This idea was further elaborated on by Donald Hebb, who made it famous (Hebb, 1949), and it was finally demonstrated decades later by Eric Kandel and others (Antonov et al., 2003)^9^.

But it was with the arrival of Cajal’s two youngest disciples that shifted the focus of the work of the Spanish Neurological School toward Neurophysiology. After training in histological techniques and centering mainly on the peripheral nervous system (see above), in the second half of the 1920s Fernando de Castro discovered the anatomical explanation of the cardiorespiratory reflexes described by Hering and Breuer (Figure 5A: de Castro, 1926, 1928, 1981; for more recent reviews see de Castro, 2008, 2009b; González et al., 2014). Yet far from fixing a separate distribution for baroreceptors and chemoreceptors in the bloodstream, de Castro tried to corroborate his neuroanatomical findings physiologically. He developed elegant but complicated approaches to show that chemoreceptors in the carotid bodies respond to changes in the chemical composition of circulating blood, combining nerve anastomosis with his grounding in nervous system regeneration (the complexity of these approaches are summarized in de Castro, 2009b; González et al., 2014). Accordingly, he performed extremely risky and lengthy experiments, suffering delays due to the political changes in Spain and the outbreak of the Spanish Civil War (1936–1939). As a result, Fernando de Castro was beaten in the race to find physiological proof for arterial chemoreceptors, a race won by the Belgian physio-pharmacologist Cornelius Heymans who was awarded the Nobel Prize in Physiology or Medicine in 1938 for these discoveries (de Castro, 2009b). From the foundations of his previous work on the structure of peripheral ganglia in normal and pathological conditions (recently reviewed in de Castro, 2016), Fernando de Castro launched his other main research line, studying the synaptic organization of the sympathetic ganglia as a means to explore the sympathetic organization of the brain (de Castro, 1937, 1942, 1950, 1951).

But it was Cajal’s youngest direct disciple, Rafael Lorente de Nó, who developed the most influential career as a neurophysiologist, completing the shift of Cajal’s School toward physiology. Trained as an expert neuroanatomist (see above) and representing a pioneer in the study of the acoustic system (Lorente de Nó, 1922, 1923), Lorente was recruited by the Nobel prize winner Robert Barany to work with him in Sweden on the physiology of the audio-vestibular system. The results of this long collaboration were published in different languages between 1925 and 1928 (for details see: Espinosa-Sánchez et al., 2019) and in a highly relevant book in the field (Lorente de Nó, 1928). Different neuroanatomical and neurophysiological aspects of current Neuro-Otology have been attributed to this singular Spaniard, who moved to the United States in 1931 after 11 months working as a clinical otorhinolaryngologist in the North of Spain (Lorente de Nó, 1931, 1932, 1933a,b,c; for a recent review of the corpus of Rafael Lorente de Nó in the context of this clinical specialty see Espinosa-Sánchez et al., 2019)^10^. It was during his second sojourn in the States, at The Rockefeller University in the 1940’s and 50’s, when Lorente de Nó became one of the most influential names in Neurophysiology. Working with motor neurons, Rafael Lorente de Nó was the first to characterize the synaptic delay, the refractory period and the spatio-temporal summation of nerve impulses, producing a bout of influential papers (Lorente de Nó, 1935a,b,c). With a strong mathematical background, Rafael Lorente de Nó also described the “recurrent neuronal circuits” in the neocortex, the first evidence of functional feedback in the physiology of the CNS (Lorente de Nó, 1938a). This was undoubtedly a fundamental concept for the later development of modern cybernetics, the consequences of which have been very recently reviewed in detail in the context of our current digital era (Espinosa-Sánchez et al., 2019).

Lorente de Nó’s “Cajalian” neuroanatomical training was fundamental for him to become the first to conceive a columnar organization of the brain and its fundamental influence on this organs function (Figure 5B: previous works were compiled in Lorente de Nó, 1938b). This was well before the first articles of Vernon Mountcastle in the field and of course, three decades before David Hubbel and Torsten Wiesel, pupils of Mountcastle, were awarded the Nobel Prize in Physiology or Medicine 1981 *“for their discoveries concerning information processing in the visual system”* (Nobel Prize Database)^11^. Rafael Lorente de Nó reached the zenith of his international recognition with his famous “A study of nerve physiology,” published in two consecutive papers of more than 1,000 pages (Lorente de Nó, 1947a,b). Although 1,000 pages of hard electrophysiology take time to read and digest, he was almost immediately nominated for the Nobel Prize in 1949 and 1950 by American institutions^12^. However, the strong and scientifically pugnacious personality of Lorente de Nó seems to have resulted in more silence than recognition in the 50 odd years that passed since he was recognized as a figure in Neurophysiology. The international scientific community, especially the Anglo-Saxons, rallied against Lorente’s refusal to accept the chemical basis of synaptic transmission and even more flagrantly, his rejection of the ionic nature of nerve impulses proposed by Allan L. Hodgkin and Andrew F. Huxley in a tremendous series of papers published between April and August 1952 in The Journal of Physiology^13^. Indeed, Lorente de Nó received his last nominations for the Nobel Prize in 1952 and 1953, both from European institutions, coinciding with Hodgkin and Eccles receiving their first nomination in 1953^14^. Once retired in 1972, as emeritus professor at UCLA, Lorente de Nó returned to his roots to compile a tome incorporating all his observations on the structure and function of the acoustic system (Lorente de Nó, 1981).

## The Contribution of the Spanish Neurological School to the Techniques Used to Study Neuroscience

Like almost every pioneer in Science, all the achievements reviewed here were based on new techniques and/or their application to new fields. It is well known that Santiago Ramón y Cajal learned the “reazione nera” (the Golgi method) from the Spanish neuropsychiatrist Luis Simarro, born in Rome and with deep intellectual and scientific roots in the last quarter of the XIXth century of Italy. Simarro had learned this histological technique from Camillo Golgi himself. Cajal, first in Barcelona and then in Madrid, perfected this capricious histological procedure in order to systematically study the fine organization of the nervous system. However, he also developed some other technical approaches to favor the application of this method, the first being the so-called “ontogenetic method” (see above), a simple but intelligent approach that allowed Cajal to achieve important contributions to our current understanding of the histology and anatomy of the nervous system (Ramón y Cajal and de Castro, 1933; de Castro et al., 2007; Merchán et al., 2016).

Nicolás Achúcarro’s main technical contribution was the introduction of the tannine-ammoniacal silver nitrate method (Achúcarro, 1911b), which was later modified by his student and successor del Río-Hortega (1916). The providential description of silver lithium carbonate impregnation, with the precisely measured formalin-ammonium bromide fixation, allowed the latter to describe the glial components as the “third element” of the CNS (del Río-Hortega, 1918), undoubtedly representing a transcendental contribution to Histology. These brief allusions highlight perhaps the most outstanding contributions to Histology by Cajal’s School. The number of methods and the relevant modifications to existing methods made by Cajal and his disciples is huge. Santiago Ramón y Cajal was deeply concerned about the future of all these technical and intellectual contributions to Science, mainly because most of them were published in Spanish. Thus, in his final years he chose one of the most skilled histologists from among his collaborators, Fernando de Castro, to systematically compile a single tome that included the techniques established at the Laboratorio de Investigaciones Biológicas over decades. This compilation that was finally published in 1933 and its English translation was only recently made available (Ramón y Cajal and de Castro, 1933; Merchán et al., 2016). In both the original and the translated versions the reader can find the vast number of recipes that Cajal and his disciples used to fix, cut and stain the nervous system.

As a further example of the technical contributions of this “Escuela de Madrid” beyond Histology, Rafael Lorente de Nó synthesized tetraethylammonium (TEA: Lorente de Nó, 1948), a compound universally used today to block potassium channels in electrophysiological studies.

## Epilogue

**FIGURE 6:**
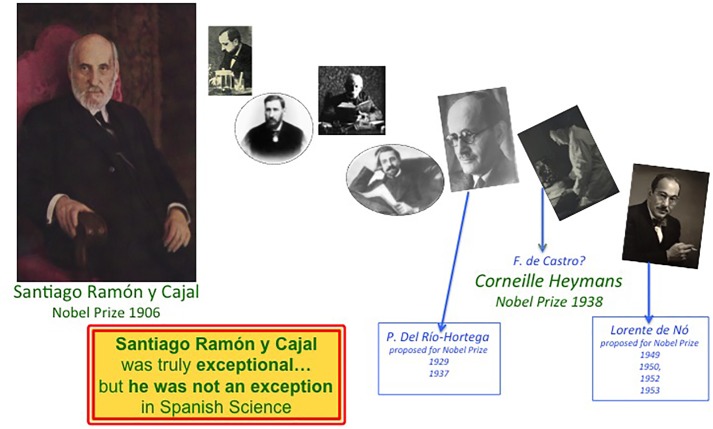
Cajal was a truly exceptional Scientist but he was not an exception for Spanish Science. The career of Cajal and of the different members of the Spanish Neurological School as they benefitted from their time with the Nobel Prize in Physiology or Medicine. While only the Maestro was awarded this prize in 1906, Río-Hortega, de Castro and Lorente de Nó were close to receiving a Nobel prize. The History of Spanish Science would be dramatically different if one of these disciples, or all of them, would also have been awarded a Nobel prize.

In this work, we have tried to systematically summarize the contributions of the Spanish Neurological School. The figure of Santiago Ramón y Cajal is universally recognized as one of the most important figures in the History of Science, yet the contributions of his main disciples (Tello, Achúcarro, del Río-Hortega, de Castro and Lorente de Nó) remain largely forgotten, despite the recent essays published to redress this balance (de Castro, 1981; Andrés-Barquín, 2002; De Carlos and Pedraza, 2014; de Castro and Merchán, 2016). Together with the School that flourished around the figure of Louis Pasteur in Paris at the end of the XIXth century, the Spanish Neurological School (also known as Cajal’s School and the Madrid School) is one of the most fruitful examples of a scientific school in Biomedicine. In the words of Charles S. Sherrington (1857–1952 and Nobel Prize 1932 in Physiology or Medicine):

“*It is no exaggeration* […] *to say that he* [Cajal] *with his pupils, especially Achúcarro, Hortega, and de Castro, opened a fresh era of knowledge*. […] *It had been an early ambition for him to find a Spanish school in science. Never has anyone stated out on a great research more single-handed than at his beginning did he. But as the years went by, if ever man had a school it was Cajal; a school of colleagues and pupils*” (Sherrington, 1935). Indeed, far from diminishing the figure of the founder, the school enhanced his importance by adding to his capacities that of recruiting, training and inspiring brilliant scientists, expanding and extending the contributions of Cajal for a further 50 years (Figure 6). Altogether, make Madrid the Mecca to learn how to approach the study of the structure and function of the nervous system between 1900 and 1936, when the Spanish Civil War disseminated its members: Lafora, Río-Hortega and most of his direct disciples exiled; Villaverde was assassinated in the revolutionary Madrid; Tello and de Castro were degraded because of their liberal ideas and were allowed to continue active in research but they suffered serious limitations; Domingo Sánchez and Pedro Ramón y Cajal were already quite old… Finally, I want to emphasize that most of the main contributions of this group of neuroscientists remain relevant today (de Castro and Merchán, 2016). Indeed, the revolutionary work by Santiago Ramón y Cajal, as well as that of his main disciples, prompted the United Nations Educational, Scientific and Cultural Organization-UNESCO in 2017 to include the archives of Santiago Ramón y Cajal, his brother Pedro, Pío del Río-Hortega, Fernando de Castro and Rafael Lorente de Nó in one of its programs of Human Heritage because *“these archives are essential to study the history of the discoveries and theories that conduct to the present comprehension of the human brain in its double aspect, anatomical composition (individual cells) and physiological properties (formation of circuits and nerve impulse propagation)”^15^.*

## Author Contributions

FdC is the single author of the entire text and figures. Regarding the latter, some of them have been adapted from previous works, as pertinently detailed in the figure legends.

## Conflict of Interest Statement

The author declares that the research was conducted in the absence of any commercial or financial relationships that could be construed as a potential conflict of interest.

## Footnotes

1 The family name of was Ramón (father’s surname) Cajal (mother’s surname), and it should be considered normal if the first surname was chosen for its general use. Besides other reasons, it seems that Cajal decided to use his maternal surname (Cajal) because the renowned German histologist August Köelliker used to do it.

2 The first volume of Cajal’s memories (“Recuerdos de mi vida” – Recollections of my life), subtitled “Mi infancia y juventud” (My childhood and youth) was published in 1901 and it was later complemented by the addition of a second section (“Historia de mi labor científica” – The story of my scientific work). The complete edition that combined these two parts was first published in Spanish in 1923, always with the common title “Recuerdos de mi vida.”

3 The 3 civil wars held in Spain in the XIXth century (1833–1840; 1846–1849 and 1872–1876) were collectively known as the Carlist wars, fought between the liberals who supported queen Isabel II (a mere girl when her father Fernando VII died) and his son Alfonso XII (during the third war), and the absolutists who originally supported the uncle of Queen Isabel II (the royal infant Carlos, brother of Fernando VII). The rebellion of the absolutists was mainly supported in the Basque Country and Catalonia. Indeed, Cajal mostly participated in the Medical Corp of the liberal army at the front in Catalonia, obviously in the 3rd Carlist War. Cuba was one of the first American territories to come under the control of the Spanish Monarchy. The fight for Independence by Cuban Spaniards started when Queen Isabel II was deposed and the first Republic was established in Spain. This political event gave rise to the Guerra Grande (1868–1878), which extended into the Guerra Chiquita (1879–1880) and the Cuban War of Independence (1895–1898), which culminated in the first international intervention by the United States and the brief Hispano-American war of 1898 (with catastrophic results for Spain).

4 The term “synapse” was introduced by Charles Sherrington.

5 It is true that although influential in some of his brother Santiago’s main discoveries (see below), Pedro Ramón y Cajal worked outside of Madrid and although he maintained a “long distance” relationship, he is undoubtedly one of the main figures of the Spanish Neurohistological School.

6 When Cajal was elected member of the Spanish Royal Academy of Medicine (1907), he specifically referred to this work on degeneration and regeneration in his inaugural lecture.

7 It is important to note that Alois Alzheimer’s first description of the disease baptized with his own name dates from 1906, when he referred to a patient of his own as “Auguste D.” in a lecture.

8 It is now more frequently referred to as Lafora progressive myoclonic epilepsy or MELF.

9 In Hebb’s proposal, the phenomenon set out by Cajal was further developed by adding mechanical features taken from the recurrent or reverberant circuits first proposed in 1938 by the youngest direct disciple of Cajal, Rafael Lorente de Nó (as detailed in this article).

10 Nevertheless, many of his studies on the audio-vestibular system were not published for several decades, once he had retired, including many of Lorente’s most outstanding hand-drawings (Lorente de Nó, 1981).

11 To be precise, the Nobel Prize in Physiology or Medicine 1981 was shared by Hubbel and Wiesel, and by Roger W. Sperry “for his discoveries concerning the functional specialization of the cerebral hemispheres”.

12 Data available in the Nobel Prize Database (Nominations Archive).

13 Sir John Carew Eccles, Alan Lloyd Hodgkin and Andrew Fielding Huxley shared the Nobel Prize in Physiology or Medicine 1963 “for their discoveries concerning the ionic mechanisms involved in excitation and inhibition in the peripheral and central portions of the nerve cell membrane” (Nobel Prize Database).

14 Data available in the Nobel Prize Database (Nominations Archive).

15 http://www.unesco.org/new/en/communication-and-information/memory-of-the-world/register/full-list-of-registered-heritage/registered-heritage-page-1/archives-of-santiago-ramon-y-cajal-and-the-spanish-neurohistological-school/
